# Evaluating the quality of social media content on metabolic dysfunction associated steatotic liver disease: An experience from a lower middle-income country

**DOI:** 10.1371/journal.pone.0343573

**Published:** 2026-03-13

**Authors:** Madunil Anuk Niriella, Indeewari Prathibha Wijesingha, Krishanni Prabagar, Dhanushi Abeynayake, Hiruni Jayasena, Piyal Rangana Herath, Anuratha Kajendran, Vithiya Rishikesavan, Karthiha Balendran, Tiloka de Silva, Arjuna Priyadarshin De Silva, Hithanadura Janaka de Silva

**Affiliations:** 1 Faculty of Medicine, University of Kelaniya, Ragama, Sri Lanka; 2 Ambilipitiya District General Hospital, Ambilipitiya, Sri Lanka; 3 Faculty of Medicine, Sir John Kothalawala Defence University, Dehiwala- Mount Lavinia, Sri Lanka; 4 Polonnaruwa Teaching Hospital, Polonnaruwa, Sri Lanka; 5 Jaffna National Hospital, Jaffna, Sri Lanka; 6 Kaluthara Teaching Hospital, Kaluthara, Sri Lanka; 7 Faculty of Medicine, University of Jaffna, Jaffna, Sri Lanka; 8 Faculty of Business, University of Moratuwa, Katubedda, Sri Lanka; Universita degli Studi della Campania Luigi Vanvitelli Scuola di Medicina e Chirurgia, ITALY

## Abstract

**Introduction:**

Metabolic dysfunction associated with steatotic liver disease (MASLD)/non-alcoholic fatty liver disease (NAFLD) represents a significant public health concern. Social media (SoMe) increasingly influences health perceptions in lower-middle-income countries, with one-third of Sri Lanka’s population using SoMe for health information. Assessing MASLD content quality on SoMe is therefore important.

**Aims & methods:**

This cross-sectional study assessed accuracy, completeness, and quality of MASLD content across Facebook, YouTube, TikTok, Instagram, and X in Sinhala, English, and Tamil from Sri Lanka (January 2005-December 2024). Board-certified gastroenterologists independently reviewed posts using standardised scales for accuracy (0–3), completeness (0–5), and global quality score (GQS) (0–5). Posts were categorised by source profile and content type, with user interactions analysed.

**Results:**

Analysis included 289 posts: 158 (54.7%) YouTube, 101 (34.9%) Facebook, 14 (4.8%) TikTok, 11 (3.8%) X, 5 (1.7%) Instagram. Languages: 214 (74.0%) Sinhala, 54 (18.7%) Tamil, 21 (7.3%) English. Content sources: undisclosed identity (36.0%), non-healthcare persons (26.0%), healthcare professionals (22.1%), alternative healthcare professionals (14.2%), healthcare institutions (1.7%). Health promotion (61.9%) was the predominant content type. Mean accuracy was 1.78/3 (59.3%), with healthcare professionals scoring highest (2.35/3, 78.5%) versus others (51.0–55.1%; p < 0.001). Completeness averaged 2.1/5 (42%), with English content scoring higher than Sinhala and Tamil. GQS averaged 2.4/5 (48.4%). 82% of posts were classified as “Rotten” (<60% score for each metric). Facebook and YouTube showed significantly higher completeness and GQS (p < 0.05). User engagement metrics showed no correlation with content quality.

**Conclusion:**

Most SoMe content originated from non-healthcare sources. Healthcare professionals delivered the most accurate content. Facebook and YouTube showed relatively higher content quality scores, though comparisons are limited by the small number of posts from other platforms. Overall quality remained suboptimal across platforms, with 82% failing adequate standards. User engagement didn’t correlate with quality. These findings highlight the need for improved quality control and health literacy initiatives for MASLD information on SoMe platforms.

## Introduction

Hepatic steatosis (or fatty liver) is a condition characterised by intrahepatic triacylglycerol accumulation of at least 5% of liver weight or 5% of hepatocytes containing lipid vacuoles [1]. Metabolic dysfunction-associated steatotic liver disease (MASLD), previously known as non-alcoholic fatty liver disease (NAFLD), is the most common cause of hepatic steatosis. MASLD is also the most common chronic liver disease worldwide [2]. Other causes of hepatic steatosis include alcoholic fatty liver disease, inborn errors of metabolism, acquired metabolic disorders, viral infections, and drug-induced [3,4].

In Sri Lanka, MASLD and alcohol-related liver disease predominates [5]. Community-based studies conducted in Sri Lanka have shown that approximately 33% and 18% of adults in the urban and rural populations, respectively, have MASLD. A longitudinal follow-up study revealed a dramatic increase in prevalence to nearly 66% over 7 years in the same urban cohort, with an annual incidence rate of 6.6% [5].

Social media (SoMe) is defined as “Internet-based channels that allow users to opportunistically interact and selectively self-present, either in real-time or asynchronously, with broad and narrow audiences who derive value from user-generated content and the perception of interaction with others” [6]. SoMe originated as a tool for connectivity and networking through creating and sharing information online [7]. The first platform, similar to present-day SoMe sites, originated in the late 1990s. Since then, many social networking sites (SNS) have emerged [8]. The exponential growth of SoMe has enabled its extension to access and disseminate health information [9]. It ranges from seeking information about diseases and public health issues to gaining social and emotional support from peer-to-peer interactions [10]. The way people use SoMe differs according to their age group. However, over 50% of people in all age groups used SoMe to find available management and treatment information [11].

By January 2023, 7.2 million Sri Lankans were using SoMe [12]. Facebook and YouTube had 6.5 million and 7 million users, respectively. Other popular SoMe platforms like Instagram, LinkedIn, and Twitter had 1.8 million, 1.4 million, and 373,000 Sri Lankan users, respectively [12].

The ability of SoMe to present statistical information more interactively makes it more appealing to users, as users can post user-generated content, which often makes it difficult to differentiate between the creator and the consumer of health-related information [13]. There has been an increase in the number of patients and doctors who use SoMe for health-related purposes in both low- and middle-income countries, which has brought positive impacts related to disease surveillance, communication, and exchanging information [14]. However, using SoMe for health information has its drawbacks. Targeting users with harmful health material, displaying unhealthy behaviour, and psychological impacts from accessing unnecessary health information were some of the safety concerns in accessing SoMe for health information [15].

Poor quality of information and the potential to provide misinformation are two of the biggest concerns when using SoMe for health-related purposes [14]. A few studies are addressing SoMe content on MASLD from Asia. A survey done in Korea, analysing 47 websites related to MASLD, showed that most of them needed significant quality improvement. Though websites maintained by institutions like the government, hospitals, universities, etc., are considered more reliable, the study showed no significant difference between them and websites created by other groups (pharmaceutical companies, charities, support groups, etc.). The abundance of information makes it difficult for users to differentiate which source to use [16]. Another study from China evaluating a total of 497 videos recommended that the quality of MASLD-related videos on TikTok needed improvement. Compared with videos created by science bloggers and medical institutions, videos from health professionals may provide accurate guidance on the treatment and prevention of MASLD [17]. A similar cross-sectional study evaluating the quality of MASLD of the top 100 Chinese videos on TikTok recommended that the medical information on TikTok is not rigorous enough to guide patients to make accurate judgments, and platforms should monitor and guide publishers to help promote and disseminate quality content [18].

Health-related information on SoMe should be taken with careful evaluation of the source and its credibility. YouTube is gaining popularity as a source for disseminating health information. A review done in 2015 showed that YouTube is being used to promote unscientific therapies and contains information that is not aligned with standard treatment guidelines [19]. A study analysing Facebook posts regarding the Zika virus during a public health crisis showed that posts containing misinformation became more popular than those containing accurate information [20]. However, a study analysing TikTok posts about liver diseases showed that posts containing misinformation had a significantly smaller number of reactions when compared to posts containing accurate information [21].

The present study aims to assess the quality of content on SoMe posts containing MASLD material originating from Sri Lanka. Our primary objective was to evaluate the accuracy, completeness and quality of the content of SoMe posts related to fatty liver disease, across Facebook, YouTube, TikTok, Instagram and X, focusing on posts published in Sinhala, English, and Tamil.

## Methodology

This was an observational study to evaluate the accuracy, completeness and quality of SoMe content related to fatty liver disease in Sri Lanka. To minimise algorithmic bias, a new Gmail account was used to create fresh profiles on Facebook, YouTube, TikTok, Instagram and X, ensuring that the data collected reflected unbiased, public-facing content. The search targeted all posts published between 1^st^ January 2005–31^st^ December 2024, containing one or more of the following keywords: “fatty liver,” “fatty liver disease,” “NAFLD,” “අක්මාවේ තෙල්” (Sinhala), and “கொழுப்பு லிவர்”, “கொழுப்பு கல்லீரல்” (Tamil). Posts were eligible for inclusion if they were in Sinhala, Tamil, or English, originated from Sri Lanka, and contained at least one of the target keywords. Posts were excluded if they fell outside the specified date range or could not be verified as originating from Sri Lanka.

A two-component scoring tool developed by Lim et al. was used to assess i) the accuracy (3-point Likert scale) and ii) completeness (5-point Likert scale) of the SoMe posts [22]. The Global Quality Scale (GQS) used by Bernard et al. was also utilised in the study to rate each response with a 5-point Likert scale to evaluate the quality and usefulness of the SoMe Posts [23]. We selected our metrics on the basis that the GQS has been widely used in evaluating online health information quality, while the three metrics together provide both granular assessment (accuracy, completeness) and holistic evaluation (quality). The three metrics used in our study evaluate distinct but complementary dimensions of SoMe posts’ quality.

Each post was independently reviewed by three board-certified gastroenterologists with clinical expertise and a special interest in hepatology against the three metrics of accuracy, completeness and GQS. Accuracy was evaluated as per accepted medical knowledge, such as the definition, epidemiology, and progression of fatty liver disease, as well as management strategies like lifestyle modifications, pharmacological treatments and non-pharmacological approaches based on the most recent guidelines, such as the AASLD 2023 guideline [24]. Completeness and GQS score were at the discretion of the reviewer. The reviewers also received a separate document with guidelines for reviewers and a common briefing to ensure uniformity of the review process.

The average standardised score across reviewers was computed for each of the three key dimensions for each post. Posts were categorised as “Fresh” if they scored 60% or higher on all three metrics (accuracy, completeness and quality) or “Rotten” if otherwise.

Additionally, posts were categorised according to the source profile and content type. Health institutions (HI) (a third-party organisation like a government, university, hospital, research group, etc.), healthcare professionals (HP) (a person practicing clinical care, research or education), alternative healthcare professionals (AHP) (a person practicing indigenous or alternative medicine), non-healthcare persons (NHP) (a person with fatty liver disease or any other individual that is not a healthcare professional), and undisclosed identity (UI) (a post that belongs to a page/channel but does not provide information about the author/creator) were the categories assigned to the source profile. The type of content was categorised according to its main objective as assessed by the researchers, which included product endorsement, which supports the quality, efficacy, or branding of particular interventions or supplements, health education (HEd), health promotion (HProm), and health education and health promotion (HEd & HProm), which empower people to take charge of their health.

User interaction data were collected from each platform, including likes, views, shares, and comments, depending on the platform. For Facebook, it was likes and comments, for YouTube, it was views, likes and comments, for X, it was views, for Instagram, it was likes, and for TikTok, it was views, likes and shares.

### Dataset and compliance statement

The dataset comprised 289 publicly accessible social media posts collected from Facebook, YouTube, TikTok, Instagram, and X between January 2005 and December 2024. Variables extracted included post content, platform type, publication date, language, source category, content type, and user engagement metrics. All data were anonymized, and no personally identifiable information was recorded. Data collection and analysis methods complied with the terms of service and data use policies of all social media platforms at the time of collection. Only publicly accessible content was included, with no attempts to access private or restricted material. Data were collected through public search interfaces and no direct interaction with social media users occurred.

### Statistical methods

To identify significant differences in mean or median content quality across different platforms and source types, one-way ANOVA and Kruskal-Wallis tests were conducted. Chi-square tests were used to compare the proportion of “fresh” vs “rotten” posts across platforms and source types. Spearman’s rank correlation was applied to assess the relationship between reviewer scores and user engagement metrics (likes and views). Inter-reviewer variation was assessed using both ANOVA and the non-parametric Extended Mantel-Haenszel test to evaluate scoring consistency across the dimensions of accuracy, completeness, and GQS. Cronbach’s Alpha and the Intra-Class correlation coefficients were also computed to evaluate consistency across reviewers. This methodology allowed for a comprehensive evaluation of the reliability and reach of fatty liver-related health information disseminated through SoMe in the Sri Lankan context.

### Disclosure of ethical statements

Approval of research protocol: N/A.

Informed consent: N/A.

Registry and the Registration No. of the study/trial: N/A.

Animal Studies: N/A.

Research involving recombinant DNA: N/A.

## Results

### Content distribution and characteristics

**Fig 1:**
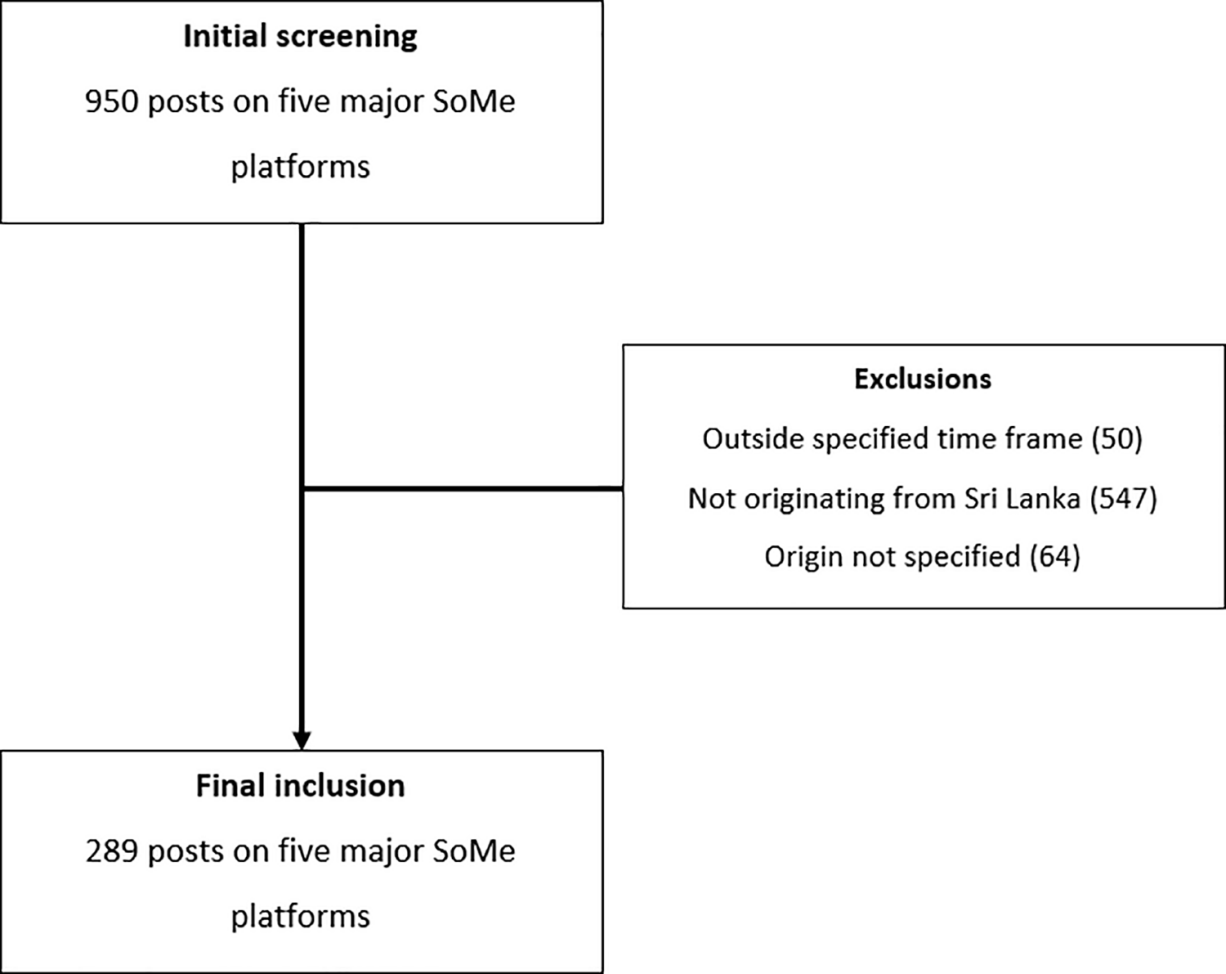
Screening for the relevant posts related to MASLD on the SoMe platforms.

Initial screening of the SoMe platforms with keywords resulted in 950 potential posts related to MASLD. After excluding irrelevant posts, a total of 289 posts across five major SoMe platforms were included in the analysis (Fig 1). YouTube emerged as the dominant platform, hosting 158 posts (54.7%) of the total content, followed by Facebook with 101 posts (34.9%). The remaining platforms showed substantially lower engagement: TikTok contributed 14 posts (4.8%), X (formerly Twitter) 11 posts (3.8%), and Instagram only 5 posts (1.7%).

Language distribution revealed a clear preference for Sinhala content, which comprised 214 posts (74.0%) of the total sample. Tamil content accounted for 54 posts (18.7%), while English posts were notably underrepresented at 21 posts (7.3%). Within platform-specific language distributions, YouTube showed the highest Sinhala content with 116 posts, followed by 28 Tamil and 14 English posts. Facebook demonstrated similar patterns with 84 Sinhala posts, 15 Tamil, and only 2 English posts. Notably, X showed an inverse pattern with 10 Tamil posts compared to only 1 Sinhala post.

### Source profile analysis

Analysis of content creators revealed concerning patterns in source credibility. The largest proportion of posts (36%) originated from undisclosed identity profiles (104 posts), raising questions about information accountability. Non-healthcare persons contributed 26% of the content (75 posts), while healthcare professionals accounted for only 22.1% (64 posts). Alternative healthcare professionals contributed 14.2% (41 posts), and health institutions had minimal representation at only 1.7% (5 posts). (Table 1)

**Table 1:** Content quality assessment by source profile.

Source Profile	n (%)	Accuracy Score†	Completeness Score‡	GQS Score‡	Fresh Content§
Healthcare Professionals	64 (22.1)	2.35 ± 0.42ᵃ	3.00 ± 0.85ᵃ	3.29 ± 0.78ᵃ	54.69%ᵃ
Alternative Healthcare Professionals	41 (14.2)	1.53 ± 0.51ᵇ	2.00 ± 0.73ᵇ	2.14 ± 0.65ᵇ	2.44%ᵇ
Non-Healthcare Persons	75 (26.0)	1.65 ± 0.49ᵇ	1.67 ± 0.62ᵇ	2.23 ± 0.71ᵇ	9.33%ᵇ
Undisclosed Identity	104 (36.0)	1.63 ± 0.55ᵇ	1.67 ± 0.68ᵇ	2.15 ± 0.73ᵇ	7.69%ᵇ
Health Institutions	5 (1.7)	–	–	–	20.0%
**Overall**	**289 (100)**	**1.78 ± 0.58**	**2.10 ± 0.82**	**2.42 ± 0.83**	**17.99%**
**p-value**		**<0.001**	**<0.001**	**<0.001**	**<0.001**

*Quality scores presented as mean ± SD with statistical significance testing*

*†Scale 1–3; ‡Scale 1–5; §Posts scoring >60% score on each metric*

*Different superscript letters indicate significant differences (p < 0.05)*

The distribution of healthcare professional content varied significantly across languages, with Sinhala posts accounting for 46 of the 64 healthcare professional posts, Tamil for 14, and English for only 4. Conversely, undisclosed identity sources were most prevalent in Sinhala content (80 posts) compared to Tamil (12 posts) and English (12 posts).

### Content type and quality assessment

Health promotion posts dominated the content landscape, representing 61.9% (179 posts) of all analysed posts. Combined health education and promotion content accounted for 17.6% (51 posts), while pure health education posts comprised 11.8% (34 posts). Product endorsements, a potentially problematic category for medical information, represented 8.7% (25 posts) of the total content. (Table 2)

**Table 2:** Platform performance and content distribution analysis.

Platform	Posts n (%)	Content Type Distribution		
		HProm	HEd + HProm	HEd
YouTube	158 (54.7)	65.8%	17.1%	11.4%
Facebook	101 (34.9)	67.3%	14.9%	5.0%
TikTok	14 (4.8)	35.7%	14.3%	35.7%
Instagram	5 (1.7)	0%	0%	80.0%
X (Twitter)	11 (3.8)	18.2%	63.6%	18.2%
**p-value**		**<0.001**		

*HProm = Health Promotion; HEd = Health Education*

*P-value is based on the Chi-squared test of independence between platform and content type*

Platform-specific content type analysis revealed that YouTube hosted the majority of health promotion posts (104 of 158 total YouTube posts), while Facebook contributed 68 health promotion posts out of its 101 total posts. Product endorsements were distributed across platforms, with Facebook showing the highest count (13 posts), followed by YouTube (9 posts).

### Quality metrics and platform performance

The overall accuracy of MASLD-related SoMe content was suboptimal, with a mean accuracy score of 1.78 out of 3 (59.3%, SD = 0.58). Completeness scores averaged 2.10 out of 5 (42%, SD = 0.82), while the Global Quality Score (GQS) reached only 2.42 out of 5 (48.4%, SD = 0.83). These metrics indicate substantial room for improvement in SoMe health information quality.

Platform-specific analysis revealed significant differences in content quality (p < 0.05). YouTube demonstrated superior performance with a mean completeness of 2.26 out of 5 (44.26%) and GQS of 2.49 out of 5 (49.83%), while Facebook achieved a mean completeness of 2.08 out of 5 (41.25%) and GQS of 2.42 out of 5 (48.45%). Other platforms collectively showed inferior performance with a mean completeness of 1.68 out of 5 (33.56%) and GQS of 2.13 out of 5 (42.67%). The median values corroborated these findings, with YouTube and Facebook showing median completeness of 2.0 out of 5 (40%) compared to 1.67 out of 5 (33.33%) for other platforms.

### Source credibility and quality correlation

Healthcare professionals produced significantly higher quality content across all evaluated dimensions (p < 0.001). Their posts achieved the highest accuracy score of 2.35 out of 3 (78.47%), compared to alternative healthcare professionals at 1.53 out of 3 (50.95%), non-healthcare persons at 1.65 out of 3 (55.11%), and unidentified sources at 1.63 out of 3 (54.27%). Healthcare professional content also excelled in completeness at 3.0 out of 5 (60%) and GQS at 3.29 out of 5 (65.73%), demonstrating statistically significant superiority across all quality domains. Alternative healthcare professionals showed a markedly lower performance with completeness of 2.0 out of 5 (40%) and GQS of 2.14 out of 5 (42.76%). Non-healthcare persons achieved completeness of only 1.67 out of 5 (33.33%) and GQS of 2.23 out of 5 (44.62%), while undisclosed identity sources performed similarly with completeness of 1.67 out of 5 (33.33%) and GQS of 2.15 out of 5 (43.08%).

### Language-based quality variations

Significant quality disparities emerged across language groups (p < 0.05). English posts demonstrated the highest completeness scores at 2.52 out of 5 (50.48%), followed by Tamil at 2.17 out of 5 (43.33%). Sinhala posts, despite comprising the majority of content, showed significantly lower completeness at 2.05 out of 5 (40.97%).

Accuracy patterns varied, with English posts achieving 2.08 out of 3 (69.31%), Tamil posts 1.93 out of 3 (64.40%), and Sinhala posts 1.72 out of 3 (57.3%). GQS scores followed similar trends: English posts scored 2.86 out of 5 (57.14%), Tamil posts 2.65 out of 5 (52.96%), and Sinhala posts 2.33 out of 5 (46.67%). Median values reinforced these patterns, with English and Tamil posts showing a median accuracy of 2.0 out of 3 (66.67%) compared to Sinhala posts at 1.67 out of 3 (55.56%). (Table 3)

**Table 3:** Language-based content analysis and quality assessment.

Language	Posts n (%)	Primary Platform	Quality Performance		
		Top Platform	Accuracy	Completeness	GQS
Sinhala	214 (74.0)	YouTube (54.2%)	57.3%	41.0%	46.7%
Tamil	54 (18.7)	YouTube (51.9%)	64.4%	43.3%	53.0%
English	21 (7.3)	YouTube (66.7%)	69.3%	50.5%	57.1%
**Significance**			Significant differences between all languages	Significant difference between English and Sinhala	Significant difference between English and Sinhala

*Differences in mean quality scores between languages tested using one-way ANOVA*

### Content “Freshness” and engagement analysis

The freshness analysis revealed alarming quality deficiencies, with 237 posts (82.01%) classified as “rotten” (scoring <60% on each metric of reviewer score). Only 52 posts (17.99%) achieved “fresh” status, indicating acceptable quality standards. Platform-specific analysis showed Facebook with 14 fresh posts (13.86% of Facebook content) and YouTube with 38 fresh posts (24.05% of YouTube content), while other platforms had zero fresh content (Chi-squared p-value 0.003).

“Freshness” analysis based on the source of the posts revealed stark differences (p < 0.001). Healthcare professionals produced 35 fresh posts out of 64 total posts (54.69%), while alternative healthcare professionals managed only 1 fresh post out of 41 (2.44%). Health institutions achieved 1 fresh post out of 5 (20%), non-healthcare persons produced 7 fresh posts out of 75 (9.33%), and undisclosed identity sources contributed 8 fresh posts out of 104 (7.69%).

Language-based freshness showed no significant differences (p = 0.755), with English posts achieving 5 fresh posts out of 21 (23.81%), Sinhala posts 37 out of 214 (17.29%), and Tamil posts 10 out of 54 (18.52%).

### User engagement and quality relationship

Contrary to expectations, user engagement metrics showed no significant correlation with content quality. Statistical analysis revealed no meaningful differences in user engagement metrics between fresh and non-fresh posts. Furthermore, correlation analyses using both Pearson’s and Spearman’s tests demonstrated no significant relationships between accuracy, completeness, or GQS scores and user engagement metrics (views or likes). These findings suggest that SoMe algorithms and user preferences do not necessarily promote higher-quality health information. (Table 4)

**Table 4:** Content Quality vs. User engagement correlation analysis.

Quality Metric	User Engagement Metric	
	Views	Likes
**Accuracy Score**	r = 0.048	r = 0.029
**Completeness Score**	r = 0.097	r = 0.130**
**GQS Score**	r = 0.035	r = 0.051
**“Fresh” vs. “Rotten” Content**		
Mean Engagement Metric (Fresh) with 95% Conf. Int.	89,843 ± 66,375	1,511 ± 1,045
Mean Engagement Metric (Rotten) with 95% Conf. Int.	74,282 ± 35,054	940 ± 431
Significance	p-value = 0.692	p-value = 0.279

*Correlation coefficients (r) using Spearman’s rank correlation*

* denotes p < 0.1, ** p < 0.05, ***, p < 0.01

*“Fresh” content is defined as a composite score of >60%*

*Significance provides the p-value of the t-test for equality of the engagement metric across “fresh” and “rotten” posts*

### Inter-reviewer reliability

The evaluation framework demonstrated robust reliability across reviewer groups. For English and Tamil posts, overall scores ranged from 53.04% to 56.15% across three reviewers, with accuracy scores between 1.85 and 2.05 out of 3, completeness scores between 2.17 and 2.36 out of 5, and GQS scores between 2.64 and 2.79 out of 5. For Sinhala posts, three reviewers showed consistent overall scores between 47.80% and 48.58%, with accuracy scores ranging from 1.62 to 1.79 out of 3, completeness from 1.86 to 2.15 out of 5, and GQS from 2.22 to 2.44 out of 5.

While some sub-component score differences were observed, likely attributed to language-specific interpretation challenges, the consistency in global scores supports the validity of the assessment methodology. This reliability strengthens confidence in the study’s quality assessments and cross-platform comparisons. (Table 5)

**Table 5:** Inter-reviewer reliability and methodological validation.

Assessment Domain	Reviewer Agreement	Content Language	Statistical Measures
	ICC	Cronbach’s α	Primary
**Accuracy Assessment**	0.728***	0.785	English/Tamil
**Completeness Assessment**	0.807***	0.836	English/Tamil
**GQS Assessment**	0.739***	0.804	English/Tamil
**Content Classification as “Fresh”**	0.790***	1.000	All languages

*ICC = Intraclass Correlation Coefficient. Values closer to 1 denote strong agreement between reviewers.*

Asterisks indicate the significance of correlation coefficient. *** denotes p < 0.01, ** p < 0.05, * p < 0.1

*Cronbach’s α > 0.7 and α > 0.8 indicate good and excellent reliability.*

## Discussion

### Principal findings and their significance

This wide-ranging analysis of 289 MASLD-related SoMe posts from Sri Lanka reveals a concerning digital health information landscape characterised by widespread misinformation and quality deficiencies. The dominance of YouTube (54.7%) and Facebook (34.9%) as primary platforms for health information dissemination, combined with the overwhelming prevalence of Sinhala content (74.0%), reflects the linguistic and technological preferences of Sri Lankan health information seekers. The majority of posts, being from YouTube and Facebook, represent the trends in SoMe use in Sri Lanka at present [25].

The most alarming finding is that 82% of all analysed posts failed to meet acceptable quality standards, representing a significant threat to public health decision-making. The stark quality disparities observed across content sources underscore fundamental issues in digital health communication. Healthcare professionals, despite representing only 22.1% of content creators, consistently produced superior quality information with accuracy scores of 78.47%, completeness of 60%, and GQS of 65.73%. This contrasts sharply with the predominant undisclosed identity sources (36% of posts), which achieved accuracy scores of only 54.27%. The fact that anonymous and non-professional sources dominate the information landscape while producing poor-quality content consistently highlights a critical gap in authoritative health communication.

The language-based quality variations present particularly concerning implications for health equity. English posts demonstrated superior completeness (50.48%) compared to Sinhala posts (40.97%), despite Sinhala being the dominant language. This disparity suggests that the majority of Sri Lankan health information seekers, who primarily consume Sinhala content, may be exposed to lower-quality information, potentially exacerbating health disparities.

### Contextualising findings within global literature

Our findings align with international research demonstrating the unreliability of SoMe health information while revealing unique characteristics of the South Asian digital health landscape [16–18]. The disconnect between content quality and user engagement observed in our study echoes patterns documented during the 2015 Ebola outbreak, where popular YouTube videos often lacked critical health information, demonstrating that viral content does not guarantee accuracy [26]. Similarly, COVID-19 vaccination research in rheumatic diseases confirmed that medical professionals produce significantly higher-quality content than general public contributors [27].

Cross-cultural studies from China and Korea provide a particularly relevant context for our findings. A comparative analysis of MASLD-related TikTok videos between China and the USA revealed similar patterns of medical practitioner reliability, supporting the universal nature of healthcare professional content superiority [28]. Korean research on MASLD websites found overall poor quality of online health information [16], emphasising the urgent need for institutional regulation and quality control mechanisms—a conclusion that resonates strongly with our Sri Lankan findings.

However, our study reveals unique characteristics of the South Asian digital health environment. The clear dominance of undisclosed identity sources (36%) compared to healthcare professionals (22.1%) suggests a more pronounced information authority gap than reported in Western contexts. Additionally, the significant language-based quality disparities we observed may reflect broader challenges in multilingual health communication that are less prominent in predominantly monolingual societies.

### Platform-specific implications and digital health ecology

The superior performance of YouTube and Facebook in supporting higher-quality content (mean GQS of 52.53% and 53.14% respectively) compared to other platforms (43.56%) reveals important insights about platform affordances and health communication. These platforms’ support for longer-form content and multimedia elements may facilitate more comprehensive health information presentation. The absence of fresh content on newer platforms like TikTok, X, and Instagram suggests that these platforms may be particularly susceptible to health misinformation propagation.

The platform-specific content type distributions provide additional insights into digital health communication strategies. YouTube’s dominance in health promotion content (104 of 158 posts) and Facebook’s substantial contribution (68 of 101 posts) indicate that these platforms have become primary venues for health advocacy. However, the presence of product endorsements across platforms (25 posts total, with Facebook leading at 13 posts) raises concerns about commercial influence on health information.

### The engagement-quality paradox and its implications

Perhaps the most troubling finding is the complete absence of correlation between content quality and user engagement metrics. This disconnect suggests that SoMe algorithms and user behaviour patterns do not inherently favour accurate health information, potentially creating echo chambers that amplify misinformation. The fact that healthcare professionals produce the highest-quality content but represent only 22.1% of creators, while achieving 54.69% fresh content rates, indicates a fundamental mismatch between content quality and visibility.

This engagement-quality paradox has profound implications for public health communication strategies. Traditional approaches that rely on content quality alone may be insufficient in SoMe environments where algorithmic promotion and user engagement drive content visibility. The finding that even high-quality medical professional content does not necessarily achieve higher engagement suggests that healthcare communicators must adapt their strategies to the unique dynamics of SoMe platforms.

### Methodological strengths and limitations

Our study’s approach, encompassing multiple platforms and languages with evaluation by board-certified gastroenterologists, provides a robust insight into South Asian digital health communication. The inclusion of three languages spoken in Sri Lanka enables culturally appropriate assessment of health information quality, while the multi-platform analysis captures the diverse digital health landscape.

However, there are several limitations. The data may be skewed, given majority were from YouTube and Facebook. The current use of each of the SoMe platforms within Sri Lanka was not considered and corrected for in this study. The restriction to Sri Lankan content limits generalizability to broader South Asian populations, though it provides valuable insights into a previously understudied topic in the region. The necessity of using different reviewer panels for different languages, while methodologically sound, may introduce subtle evaluation variations. The absence of a formal scoring trial before assessment and the lack of multiple comparison correction represent a methodological limitation that future studies should address through comprehensive inter-rater reliability protocols.

Additionally, our focus on MASLD-specific content may not fully represent the broader digital health information landscape. Future research should expand to include other prevalent chronic diseases and examine longitudinal changes in content quality and user engagement patterns.

### Public health implications and recommendations

The findings of this study, we believe, have immediate implications for public health policy and digital health literacy initiatives in Sri Lanka and other LMIC. The predominance of poor-quality health information from unverified sources, combined with the limited presence of healthcare professionals on SoMe platforms, creates an urgent need for systematic intervention.

Healthcare institutions must prioritise digital engagement strategies that leverage the superior content quality capabilities of medical professionals while addressing the engagement-quality paradox through targeted SoMe training and platform-specific content optimisation. The development of verified healthcare professional networks on major platforms could provide audiences with easily identifiable sources of reliable health information.

Regulatory oversight mechanisms, potentially coordinated through the Ministry of Health and the Health Education Bureau, should establish guidelines for health information quality assessment and verification systems. These initiatives should specifically address the language-based quality disparities observed in our study, ensuring that health information in all local languages meets acceptable standards.

### Future research directions

Future investigations should expand beyond a single-disease focus to examine broader patterns of health misinformation across multiple conditions and platforms. Longitudinal studies tracking changes in content quality and user engagement over time would provide valuable insights into the effectiveness of intervention strategies. Additionally, user-focused research examining health information-seeking behaviours and the impact of poor-quality information on health decision-making would complement our content-focused findings.

The development and validation of automated content quality assessment tools could enable real-time monitoring of health information quality across platforms and languages. Such tools would be particularly valuable for health authorities seeking to identify and address misinformation rapidly.

Research into effective strategies for improving the visibility and engagement of high-quality health content, while maintaining scientific accuracy, represents a critical frontier in digital health communication. Understanding how to leverage SoMe algorithms and user behaviour patterns to promote evidence-based health information could transform public health communication effectiveness.

## Conclusion

This study provides the first broad analysis of MASLD-related SoMe content in Sri Lanka, revealing a digital health information landscape characterised by widespread quality deficiencies and concerning gaps in authoritative health communication. The findings underscore the urgent need for coordinated public health interventions that address both content quality and the fundamental mismatch between information reliability and user engagement in SoMe environments. The implications extend beyond Sri Lanka, offering valuable insights for digital health communication strategies in multilingual, resource-constrained contexts globally.

## Supporting information

S1 File Combined summary datasheet for SoMe posts. (RAR)
